# Signal propagation in LOV-based multidomain proteins: time-resolved infrared spectroscopy reveals the complete photocycle of YF1 and PAL

**DOI:** 10.1039/d5cp03982g

**Published:** 2026-01-08

**Authors:** Raoul E. Herzog, Philipp Janke, Paul M. Fischer, Philipp J. Heckmeier, Chongyao Wei, Probal Nag, Sina J. Hartmann, Matthias Mulder, Fabienne Stierli, Jörg Standfuss, Igor Schapiro, Peter Hamm

**Affiliations:** a Department of Chemistry, University of Zurich Winterthurerstrasse 190 CH-8057 Zurich Switzerland raoul.herzog@chem.uzh.ch peter.hamm@chem.uzh.ch; b Fritz Haber Center for Molecular Dynamics Research Institute of Chemistry, The Hebrew University of Jerusalem Jerusalem 9190401 Israel; c Department of Physics, TU Dortmund University Otto-Hahn-Str. 4 DE-44227 Dortmund Germany igor.schapiro@tu-dortmund.de; d Laboratory of Biomedical Research, PSI Center for Life Sciences Forschungsstrasse 111 CH-5232 Villigen PSI Switzerland; e Research Center Chemical Sciences and Sustainability, University Alliance Ruhr DE-44801 Bochum Germany

## Abstract

Light-oxygen-voltage (LOV) domain proteins represent a versatile class of photoreceptors capable of regulating a wide range of light-dependent biological functions. While a lot of studies have focused on the photochemistry of LOV domains, the mechanisms of signal generation and propagation in multidomain LOV proteins remain incompletely understood. Here, we investigated two multidomain proteins, using time-resolved infrared spectroscopy. The measurements resolve the entire photocycle dynamics from picoseconds to hours and uncover distinct patterns of local and global structural responses. The two multidomain proteins under study, YF1 and PAL, exhibit nearly identical dynamics during excitation and intersystem crossing on the nanosecond timescale, reflecting conserved local interactions between the chromophore and its highly conserved binding pocket. Multiscale simulations attribute minor spectral differences in this regime to a phenylalanine residue located near the chromophore present only in one of the two LOV domains. The similarities, however, end at the microsecond timescale, where adduct formation already involves global structural adaptations. By experimentally isolating the response of the histidine kinase effector domain in the synthetic photoreceptor YF1, we show that major structural adaptions of the effector domain occur concurrently with cysteine-adduct formation and that the Jα-helix putatively mediates unidirectional communication between domains. In PAL, light-induced opening of the RNA binding site during the adduct formation is additionally followed by a subsequent rearrangement in the distal PAS domain after 3 s. This highlights the pivotal yet distinct roles of the Jα-helix in signal transmission, which depend on the domain topology. Ultimately, our study not only deepens the current understanding of signal transduction in full-length LOV proteins, but also contributes to the fundamental framework for the future application of LOV domains in optogenetic engineering.

## Abstract 2

Using transient IR spectroscopy, we track the complete photocycles of the LOV-based multidomain proteins YF1 and PAL across 15 orders of magnitude in time, revealing their distinct signal transduction pathways.

## Graphical abstract

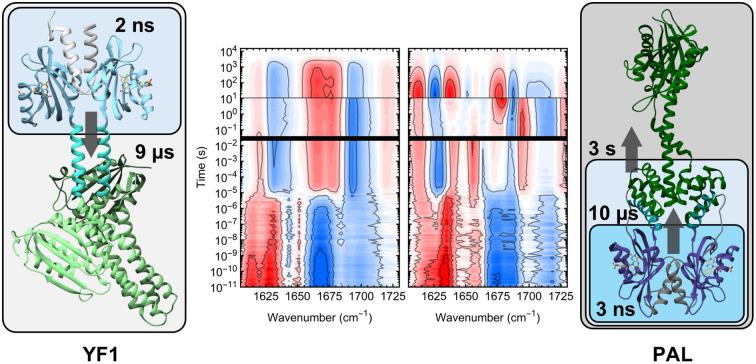

## 1 Introduction

**Fig. 1:**
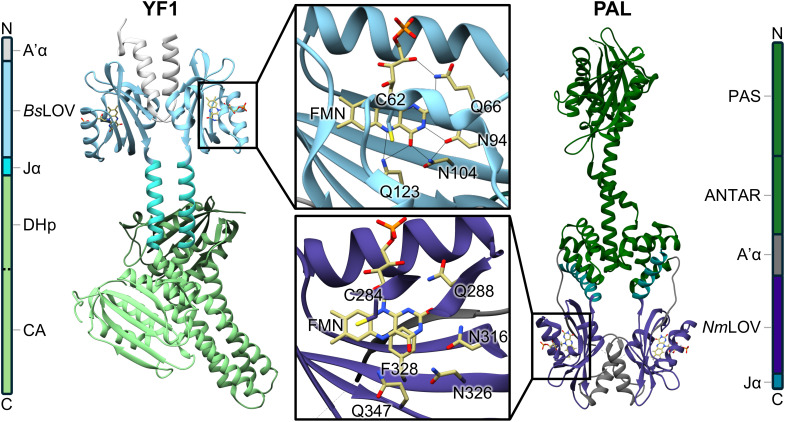
Domain architectures and crystal structures of the studied proteins YF1 (PDB: 4GCZ^9^) and PAL (PDB: 6HMJ^10^). Insets show magnified views of the chromophore-binding pocket. Relevant hydrogen bonds between the FMN cofactor and conserved residues are indicated by black lines. The insets also illustrate an exemplary partitioning of the system used in the quantum mechanics/molecular mechanics (QM/MM) calculations, where atoms shown as yellow sticks represent the QM region, and the remaining parts (shown as blue sticks and blue ribbons) correspond to the MM region.

Light-sensitive multidomain proteins play central roles in converting environmental light signals into biochemical or physiological responses. Among the various photoreceptor families, a large and diverse class employs a light-oxygen-voltage (LOV) domain as the photosensory unit.^1^ LOV domains bind flavin mononucleotide (FMN) as chromophore in a highly conserved hydrogen-bonding network (Insets of Fig. 1), enabling light-dependent control over a wide range of biological functions.^1,2^ Exposure to blue light leads to excitation of the FMN to the singlet excited state (^1^FMN*), which subsequently decays on a nanosecond timescale to the triplet state (^3^FMN*) *via* intersystem crossing (ISC).^3–5^ Upon decay of the triplet state (microsecond timescale), a reversible thioether bond is formed (*A*_390_) with a cysteine residue, which is highly conserved for the vast majority of known LOV domains.^2,6^ Mediated *via* hydrogen bonding rearrangements involving a “lever”-like motion of a conserved Gln residue, the light-induced signal triggers conformational changes in the LOV domain and thereby initiating downstream signaling across the multidomain scaffold, resulting in the light-adapted state (SIG).^7,8^

Signal propagation in LOV-based photoreceptors involves a complex interplay between local rearrangements in the FMN binding pocket and long-range conformational transitions that regulate effector activity.^11^ While the photochemical formation of the cysteine adduct is well established, the precise sequence of electronic and structural events leading to this reaction and the molecular processes by which local photoactivation drives large-scale functional changes across multidomain architectures remain incompletely understood.^2^ A deeper understanding of how these early photochemical events are transmitted through distinct domain architectures is essential for elucidating LOV-mediated signaling and harnessing their modular design principles. To address this challenge, we investigated the two full-length photoreceptors YF1 and PAS-ANTAR-LOV (PAL) (Fig. 1) as examples for bacterial LOV signaling, which both form homodimers^10,12^ yet differ in origin and modular composition.

The photoreceptor YF1 was engineered by genetically fusing the histidine kinase domain of FixL from *Bradyrhizobium japonicum* to the LOV domain of YtvA from *Bacillus subtilis* (*Bs*LOV), highlighting the potential of LOV domains as versatile platforms for the development of optogenetic tools.^12^ This fusion protein retains kinase activity in its dark-adapted state, closely similar to FixL, but switches to a phosphatase upon illumination. As such, YF1 serves as an excellent model for dissecting signal generation within LOV domains and its subsequent transduction to effector domains.

The photocycle of YF1 has previously been studied using time-resolved X-ray solution scattering^13^ and electron paramagnetic resonance.^14^ Concurrently with cysteine-adduct formation, the anchor points of the Jα-helices were observed to spread apart, accompanied by supercoiling of the Jα-helices, which induces rotation of the entire kinase module. After 250 ms, a slower internal rearrangement in the histidine kinase effector module has been observed, characterized by a relocation of the ‘catalytic and ATP binding’ (CA) subdomains on the ‘dimerization and histidine phosphotransfer’ (DHp) subdomains.^13^

The other system under investigation is the PAL receptor protein from the Gram-positive actinobacterium *Nakamurella multipartita*.^10^ Unlike most LOV-containing receptors, in which the LOV domain is located at the N-terminus and transmits signals *via* the Jα-helix, the LOV domain of PAL (*Nm*LOV) is located C-terminally to its PAS (Per-Arnt-Sim) and its RNA-binding ANTAR (amiR and NasR transcription antitermination regulators)^15^ domains. This inverted domain architecture presents a unique opportunity to explore alternative signal propagation mechanisms and the specific role of the Jα-helix in mediating downstream effects.

Despite the wealth of experimental and computational efforts, elucidation of the precise mechanism and kinetics of initial signal generation in the LOV domain and its propagation to the effector domain in multidomain proteins remain elusive. In this study, we provide insight into the photocycle of proteins that incorporate LOV domains using YF1 and PAL as exemplary models. To this end, we employ time-resolved infrared spectroscopy (TR-IR) in the amide I region. This spectral window is characterized by its sensitivity to changes in secondary structure as well as characteristic chromophore vibrations.^16^ Here, we present TR-IR data of the full-length proteins YF1 and PAL spanning 15 decades in time, hereby observing the entire photocycle from signal generation, over transduction, and finally to dark-state recovery.

## 2 Materials and methods

### 2.1 Protein preparation

The three samples *Bs*LOV, YF1, and PAL (Fig. S1) were heterologously expressed in *Escherichia coli*. The exact protocol was adequately adapted to achieve an optimal yield for every sample. For the generation of *Bs*LOV and YF1, we used a riboflavin synthase-depleted C41 (DE3) strain (CpXribF; manX::ribM ribC::cat-ribF; kindly provided by Tilo Mathes *et al.*,^17^) for PAL, we used a commercial BL21 (DE3) strain. Cells were grown at 37 °C until OD_600_ = 0.6 was reached. Then, expression was induced by adding 1 mM Isopropyl-β-d-thiogalactopyranosid. After induction, *Bs*LOV was incubated at room temperature, YF1 was cooled down to 18 °C, PAL to 16 °C. The expression was stopped after 20 h, cells were harvested and lysed. From the crude cell extract, the target protein, which was modified to include a poly-His tag, was isolated *via* Ni-affinity chromatography. Purity and integrity of the target protein were controlled by SDS-PAGE or mass spectrometry. For subsequent experiments, the samples were provided in individual buffers. *Bs*LOV: 50 mM Tris-HCl (pH/pD 8), 125 mM NaCl. YF1: 10 mM Tris-HCl (pH/pD 8), 10 mM NaCl, 10% (v/v) glycerol. PAL: 12 mM HEPES (pH/pD 7.4), 250 mM KCl, 250 mM NaCl, 1 mM MgCl_2_.

### 2.2 Time-resolved measurements

TR-IR spectra up to 40 ms were measured using a 100 kHz Yb-doped fiber laser/amplifier system (short-pulse Tangerine, Amplitude, France) together with an OPA (Twin STARZZ, Fastlite, France) with a subsequent frequency mixing stage in a LGS crystal as probe and reference pulses. The pump pulses were obtained by an electronically synchronized frequency doubled Ti:Sa oscillator (Spectra Physics, Milpitas, CA, USA), to give pump pulses centered at ≈420 nm with a typical energy of ≈4 µJ and a temporal full width at half maximum (FWHM) of ≈120 fs. The pump pulses were rotated by a *λ*/2 waveplate to give magic angle pump–probe polarizations. Pump and probe pulses were focused and overlapped in the sample (*ca.* 220 µm and 120 µm FWHM), while the IR-reference (120 µm FWHM) was slightly offset (*ca.* 500 µm). Probe and reference beams were transmitted through a spectrograph and detected with a 2 × 32 MCT array detector (spectral resolution of ≈4 cm^−1^ per pixel) with customized measurement electronics.^18^

The samples were kept in the dark overnight before measurements to ensure complete thermal relaxation to the dark-adapted state. Between subsequent pump pulses, the sample was exchanged using a peristaltic pump combined with the stop-flow system described in ref. 19. An additional flow path containing a back-pressure regulator was connected after the pressure reservoir, to regulate the pressure without adjusting the speed of the peristaltic pump. The setup is illustrated in Fig. S4. A flowcell with two CaF_2_ windows and a 50 µm ultrathin silicone film spacer (SILPURAN, Wacker Chemie AG, Munich, Germany) was used as sample cell.

TR-IR spectra from 40 ms to hours were measured with a commercial Vertex 80v FTIR spectrometer (Bruker Corp., Billerica, MA, USA) equipped with a N_2_-cooled MCT detector (Kolmar Technologies, Newburyport, MA, USA) in rapid-scan mode. For measurements between 40 ms and 15 s, the same stop-flowcell was used as described above to exchange the sample volume after each repetition. The experiment was repeated between 200 and 600 times and averaged to improve signal-to-noise ratio. Illumination was conducted at 447 nm using a GaN multimode laser diode (PLPT9 450D_E, Osram Opto Semiconductors), operated by a pulsed laser diode driver (LDP-V 10-10, PicoLAS) to produce 1–2 ms pulses with a typical pulse energy of 10–20 mJ. The spot size was ≈2 mm in diameter and the spectrometer data acquisition rate 160 kHz.

For measurements of the dark adaption on the minutes to hours time-scale, the acquisition rate was set to 40 kHz and a static sample cell was used, consisting of two CaF_2_ windows with a 50 µm Teflon spacer to ensure constant thickness. One of the windows was modified with a concentric groove with an inner diameter of 5 mm to prevent capillary effects of the spacer and ensure sample stability over the long-term measurement. Sample excitation was achieved by illuminating for 30 s with a 30 mW continuous wave laser at 450 nm (OXLasers, China). The time-resolution was ≈300 ms.

Using the femtosecond laser setup TR-IR data was measured up to varying maximal delay times. This enabled us to balance the signal-to-noise ratio at late delay times (binning) and early delay times (averaging) to some degree. Slight variations between obtained batches were accounted for by linear scaling at overlapping times before averaging. A full spectrum of each protein was recorded in this fashion on multiple individual days and the spectra were averaged.

The TR-IR FTIR data in rapid-scan mode between 40 ms and 15 s was corrected with a linear baseline by fitting and subtracting a linear function to the endpoints of each spectrum. The back-reaction measurements were subjected to a singular value decomposition to isolate and delete the baseline drifts occurring on the minutes to hours time-scale. To ensure retention of a complete orthonormal basis, a rotation procedure was applied to the singular value decomposition as described by Henry and Hofrichter.^20^

### 2.3 Multiscale simulations

Hybrid QM/MM calculations were performed to support the assignment of C2=O and C4=O vibrations in the IR difference spectra. These multiscale simulations were based on the experimental structures of *Bs*LOV and *Nm*LOV, which were obtained from the crystal structures of YF1 (PDB ID: 4GCZ^9^) and PAL (PDB ID: 6HMJ^10^), respectively. These protein structures are available in the resting state. The PDB files were prepared for simulation by adding missing structural information, determining protonation states and solvating it in a water box. Subsequently, an energy minimization was performed to relax the protein. In this step of the process, the employed force field was ff14SB^21^ for the protein and TIP3P^22^ for water. FMN parameters were adapted from Schneider *et al.*^23^

The QM/MM geometry optimizations and vibration analysis procedures were conducted for the ground state of the LOV domains using Gaussian16.^24^ The optimization was performed using B3LYP-D3BJ/6-31G*. The QM region included the FMN and the residues forming the binding pocket (see yellow sticks in the insets of Fig. 1).^25,26^ For calculations of the *Nm*LOV F328L mutant, the leucine that was introduced in place of phenylalanine was excluded from the QM region to retain consistency with the *Bs*LOV QM region. The vibrational calculation was performed using B3LYP-D3BJ/6-311G*, and the wavenumber axis was scaled using a factor of *α* = 0.966.

The vibrational bands are broadened by a Gaussian function with a FWHM of 16 cm^−1^ and then plotted as IR spectra in the range of the experimentally recorded spectra. The vibrational modes were assigned by visual inspection.

## 3 Results

### 3.1 Dynamics of the photocycle

**Fig. 2:**
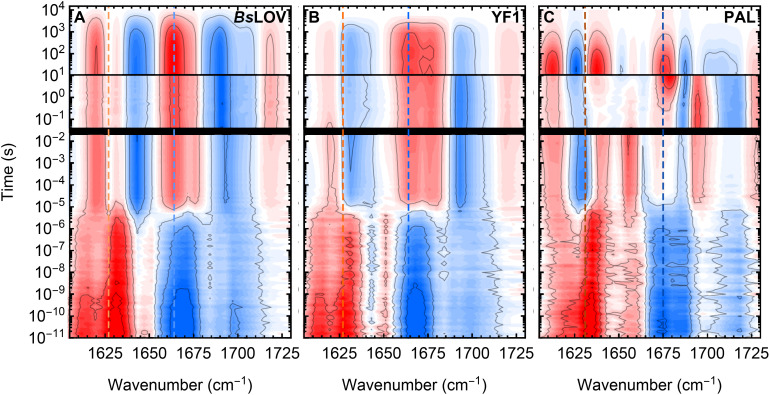
Contour plots representing the TR-IR data in the amide I region of the LOV photoreceptors *Bs*LOV, YF1, and PAL. Negative signals (blue) arise from ground state modes of the dark-adapted state which are depopulated or exhibit a decreased extinction coefficient due to electronic changes. Positive signals (red) correspond to populated modes from formed intermediates during the photocycle. The time is plotted on the y-axis and scaled logarithmically in order to reveal the exponential kinetics. The spectra obtained from individual measurements are shown combined in a single plot, separated by black lines representing the time-gaps in the acquired TR-IR data (see Materials and methods). The colored dotted lines indicate the representative kinetic traces shown in Fig. 3.

In this study, we resolve the photocycle of the full-length multidomain proteins YF1 and PAL. The experimentally obtained TR-IR spectra are shown in Fig. 2 as normalized contour plots. The spectra recorded before and after the time-gaps (black lines) exhibited variations in the relative band intensities, which we attribute to variations in the measurement procedures. Nevertheless, the spectra demonstrated satisfactory qualitative agreement.

The data were analyzed by global fitting, resulting in a sequential model approximating the TR-IR data. The fits were performed individually for the three recorded time windows. In order for this to accurately yield all kinetic constants, this requires that no processes occur during the time-gaps. This assumption seems strongly supported by the high similarity of the spectra recorded before and after the gap with the respective methods (see Fig. S3).

**Fig. 3:**
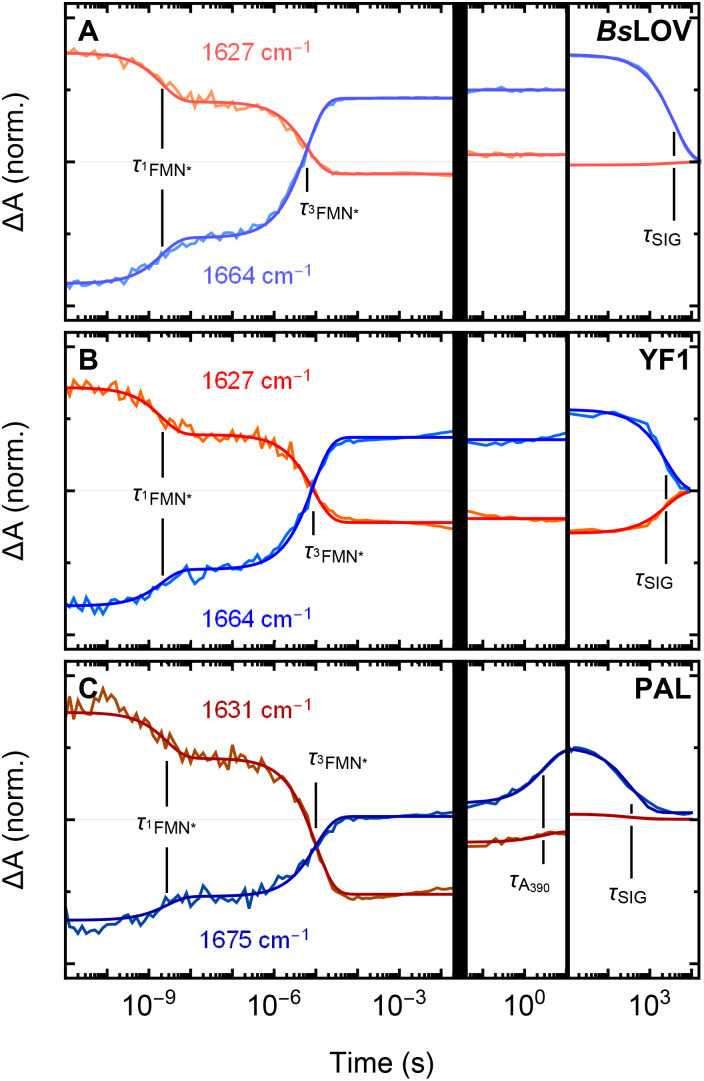
Representative kinetic traces and their respective fits from global fitting. The processes associated with the respective spectral changes are labeled.

The obtained fits and the corresponding residuals are shown as contour plots in Fig. S2. In Fig. 3, two representative kinetic traces with the respective fits for each of the proteins are shown. We found that ^1^FMN* decays with *τ*_^1^FMN*_ ≈ 2–3 ns to ^3^FMN*, followed by formation of *A*_390_ with *τ*_^3^FMN*_ ≈ 6–10 µs. The formation of the adduct state has previously been observed to involve dispersive kinetics in multiple related systems, including YtvA.^27,28^ However, our data did not indicate the presence of an additional kinetic component. Monoexponential kinetics was sufficient to accurately fit the decay of ^3^FMN*. On the millisecond time-scale, minor deviations from the fits were observed in the YF1 and PAL spectra, characterized by an unspecific offset in the majority of the amide I region. The lack of changes in specific spectral characteristics, as is evident from the fitting residuals (see Fig. S2F and I), prompted us to discard the possibility of this spectral change arising from a structural adaptation of the proteins. Hence, we attribute this to heating of the sample. PAL (Fig. 3C) undergoes an additional structural adaption to form SIG with *τ*_*A*_390__ ≈ 3 s. Finally, the dark-adapted state recovers with *τ*_SIG_ ranging between 370 and 3800 s (6 and 63 min).

### 3.2 Spectral features of the photocycle

**Fig. 4:**
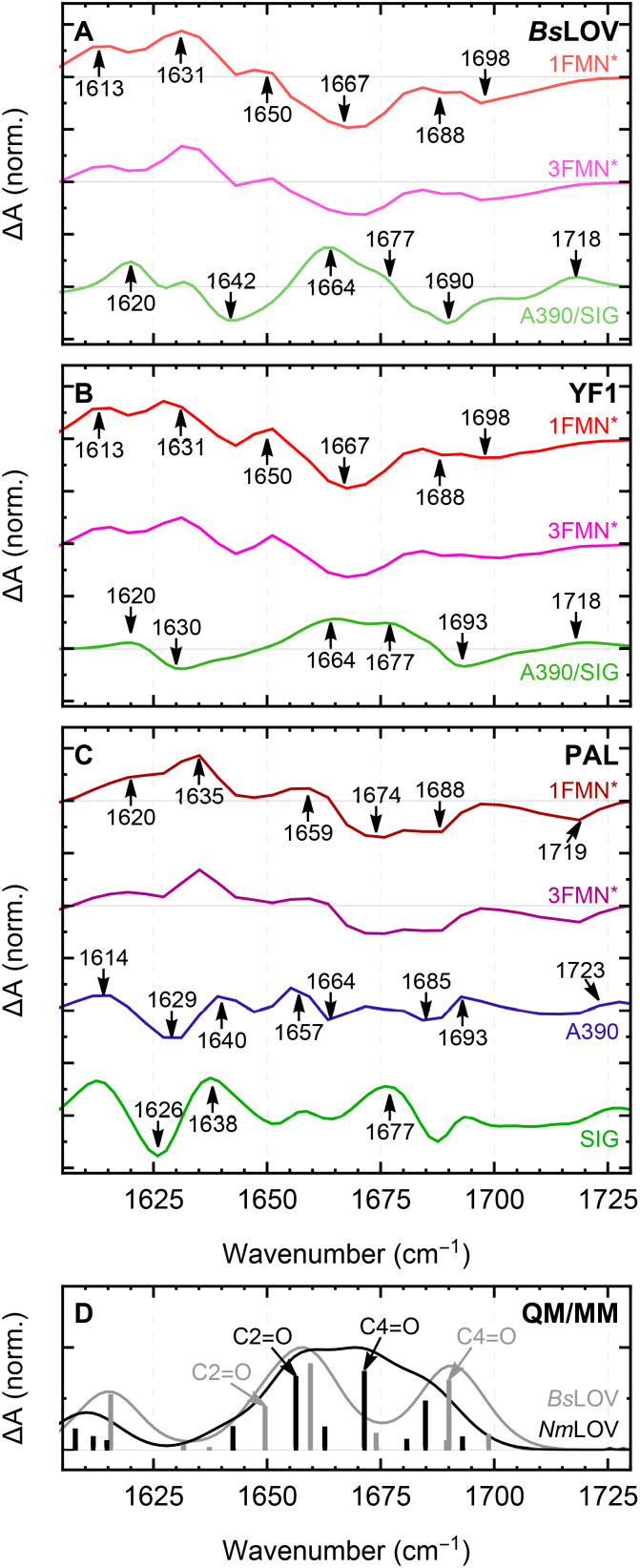
(A)–(C) Representative EADS for each photophysical state obtained from global fitting. EADS labeled as ^1^FMN*, ^3^FMN*, or *A*_390_ were obtained from global fits to the femtosecond data, EADS labeled as *A*_390_/SIG or SIG were obtained from global fits to the dark-state recovery measurements. The complete set of obtained EADS, including those associated to the same photophysical state but obtained from different measurements, are shown in Fig. S3. (D) Hybrid QM/MM calculated IR spectra of *Bs*LOV and *Nm*LOV.

The evolution associated difference spectra (EADS) that best represent the different species of the photocycles are shown in Fig. 4. The spectral features associated with ^1^FMN* of *Bs*LOV and YF1 are nearly identical, exhibiting transients at 1613 and 1631 cm^−1^, a shoulder at 1650 cm^−1^, a broad bleach at 1667 cm^−1^, and minor bleaches at 1688 and ≈1700 cm^−1^. The features agree well with those found for YtvA.^28,29^ The first EADS of PAL exhibits minor but distinct differences. The transients, the shoulder, and the broad bleach appear blue-shifted by ≈4 to 9 cm^−1^. The bleach at 1688 cm^−1^ is unaltered. The bleach at ≈1700 cm^−1^ is blue-shifted by ≈20 cm^−1^. In previous literature this bleach was assigned to C4=O.^27–29^

The transition from ^1^FMN* to ^3^FMN* is characterized mainly by a decrease in intensity of all features in the amide I region, consistent with literature.^8,27,30,31^ Since the bleaches represent the loss of ground state population, we can use their intensity ratios in the EADS of ^3^FMN* and ^1^FMN* as an estimate for the quantum yield of the ISC. This yielded ISC quantum yields (*Φ*_ISC_) between 0.6 and 0.7 for *Bs*LOV and YF1, and between 0.7 and 0.8 for PAL, respectively. Despite this method of estimation being only an approximation, the value determined for *Bs*LOV reasonably agrees with literature (0.62 (*Bs*LOV),^32^ 0.62 (YtvA)^33^ and 0.78 (YtvA)^34^).

The formation of *A*_390_ results in a significant change in the spectral characteristics of the amide I region and exhibits the most significant differences between the three systems studied. The adduct spectra of *Bs*LOV and YF1 both exhibit transients at 1620, 1664, and 1677 cm^−1^, albeit with different intensities, a bleach at 1693 cm^−1^ (*Bs*LOV) or 1690 cm^−1^ (YF1), and a transient at 1718 cm^−1^, previously assigned to the C4=O mode.^8,27^ They show differences mainly in the region around 1630 cm^−1^, where a bleach is observed for YF1, while there is a shoulder in *Bs*LOV. The bleach at 1642 cm^−1^ in the spectrum of *Bs*LOV seems to be also present, but significantly weaker in YF1. Due to the significant differences in the signals, the *A*_390_ EADS of PAL is essentially not comparable to the ones obtained for the other two systems. It is characterized by transients at 1614, 1640, 1657, 1693, and 1723 cm^−1^, as well as bleaches 1629, 1664, and 1685 cm^−1^. Finally, the formation of SIG in PAL results in a blue-shift of the 1629 bleach by about 3 cm^−1^, the bleach of the transient at 1657 cm^−1^ and finally the formation of a transient at 1677 cm^−1^.

### 3.3 Long-range signaling/interdomain communication

**Fig. 5:**
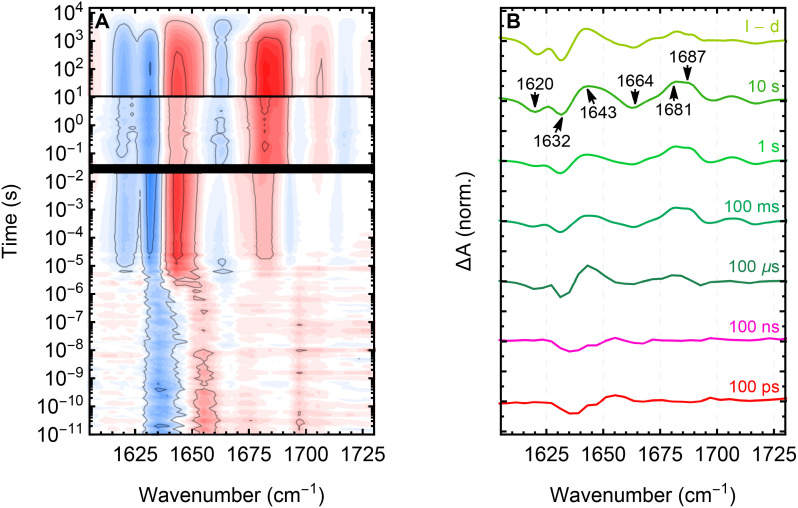
YF1-minus-*Bs*LOV double-difference spectra as a contour plot (A), and cuts at specific time points (B).

In order to elucidate the signal propagation from the LOV domain to the effector domain, we calculated the YF1-minus-*Bs*LOV double-difference spectrum. The resulting contour plot, as well as spectral traces at specific times, are shown in Fig. 5. The spectra at early times (up to ≈1 µs) exhibit only weak signals, the main features being a bleach at ≈1637 cm^−1^ and a transient at ≈1655 cm^−1^. Upon formation of *A*_390_, the double-difference spectrum reveals significant bleaches at 1620, 1632, and 1664 cm^−1^, as well as transients at 1643, 1681, and 1687 cm^−1^. Subsequently, the double-difference signal remains constant up to the dark-state recovery. The observation of the double-difference signal arising simultaneously to the decay of ^3^FMN* and staying constant afterwards strongly indicates a concurrent formation of *A*_390_ and downstream propagation of the signal. This is in perfect agreement with the result of previous X-ray solution scattering experiments, which report a splaying of the LOV domains on the microsecond timescale, causing supercoiling of the Jα-helices associated with rotation of the entire kinase module.^13^

### 3.4 Hybrid QM/MM simulations

Previous studies have established isotope labeling and quantum chemical calculations for assigning ground state and excited state FMN modes.^35,36^ To assist in the assignment of the C=O vibrations of FMN in the experimental spectra of YF1 and PAL, we carried out multiscale QM/MM simulations. We based the simulations on the crystal structures of YF1 and PAL in the resting state, from which we extracted the LOV-domains (*Bs*LOV and *Nm*LOV). Since the experimental difference spectra of YF1 and *Bs*LOV are highly similar in the bleaches that correspond to the vibrations in the resting state, we did not use the full-length YF1 structure for the present simulations.

The resulting calculated ground state spectra are shown in Fig. 4D. The computed spectrum of *Bs*LOV shows the C2=O and C4=O modes appearing as well-separated bands at 1650 cm^−1^ and 1690 cm^−1^, respectively. These vibrations roughly correspond to the 1667 cm^−1^ and 1688/1689 cm^−1^ bleaches in the experimental spectra for the ^1^FMN* states in *Bs*LOV and YF1. In contrast, the calculated *Nm*LOV spectrum features a broad band, resulting from the close overlap of the C2=O and C4=O modes centered at 1656 cm^−1^ and 1671 cm^−1^, respectively. These can be compared to two close-lying bleaches in the experimental spectrum of ^1^FMN* PAL at 1674 cm^−1^ and 1688 cm^−1^. Between the two calculated spectra, the C2=O modes coincide more closely, while the C4=O mode of *Nm*LOV is significantly blue-shifted compared to *Bs*LOV.

To elucidate the reason for these spectral differences, we searched for structural elements that could be the underlying cause. A key distinction of *Nm*LOV is the presence of a phenylalanine residue (F328), which is located close to FMN (see Fig. 1) and forms a hydrogen bond to N326 as well as π-stacking with FMN. To test its effect on the vibrational spectrum, we replaced it by leucine, which occupies the equivalent position in *Bs*LOV, and calculated the IR spectrum for the optimized geometry. The results revealed a spectrum (Fig. S6) that closely resembles that of *Bs*LOV, highlighting that F328 is responsible for the shifted FMN vibrations in *Nm*LOV.

Furthermore, the multiscale simulations show that the broad band is not solely due to the C=O vibration of the FMN. Numerous contributions come from the hydrogen-bonding partners of these two carbonyls, which are also included in the QM region. We therefore conclude that this coupling makes the experimental assignment of these vibrations difficult, even when isotope labeling is used. This is in line with recent findings by Kottke and coworkers.^37^

To understand the decrease in the intensity during the transition from the singlet to the triplet excited state, we performed quantum chemical simulations of lumiflavin. Since no crystal structure of any LOV domain is available in either of the excited states, these simulations were performed for lumiflavin in the gas phase. The lumiflavin geometries were optimized in the lowest singlet and triplet excited states, respectively. They have π–π* character in agreement with literature.^35,38^ These simulations show a downshift in the carbonyl vibrations in the singlet and triplet excited states compared to the ground state. Furthermore, there is a decrease in the intensity in the triplet state compared to that in the singlet excited states. Based on the difference in electron densities we can conclude that the character of the singlet and triplet excited states is similar (see Fig. S7).^39^ The C4=O vibration is shifted the most, but since it is a simulation in the gas phase, the hydrogen bonding partners are not included, and the shift could be different in the protein.

## 4 Discussion

**Fig. 6:**
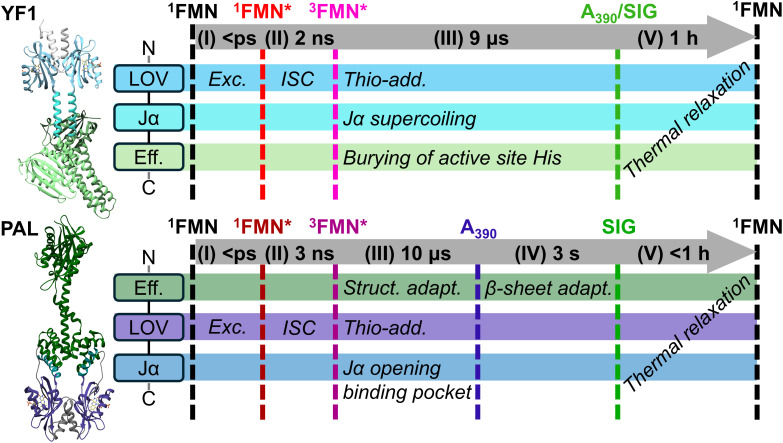
Schematic summary of the experimentally observed photocycles and the proposed processes occuring during the different steps.

Our data suggest a sequential model for the photocycle of the FMN incorporating LOV domain proteins YF1 and PAL. This model includes the following steps and timescales (summarized in Fig. 6): (I) excitation of the chromophore to ^1^FMN*, (II) ISC to ^3^FMN* on a timescale of ≈ 2 ns, (III) formation of the thioadduct *A*_390_ and concurrent long-range signal transduction on a timescale of ≈ 5 to 10 µs, (IV) in case of PAL, relaxation to the final metastable light-adapted signaling state on a timescale of seconds, and finally (V) the recovery of the dark-adapted state on a timescale of minutes to hours. In the following, we will discuss steps (II)–(V) more closely.

### 4.1 Excitation and ISC

The EADSs associated with ^1^FMN* in *Bs*LOV and YF1 (red lines in Fig. 4A and B) were found to be highly similar, yet they differed from the one observed in PAL (red line in Fig. 4C). This suggests that the spectra are shaped not only by the cofactor itself but also by the surrounding hydrogen-bonding network. The signals therefore originate from vibrational modes delocalized across this network, a conclusion further supported by our computational results. Multiscale calculations suggest that F328 interacts with FMN by π-stacking and participates in the H-bonding network.

The ISC quantum yield was found to be equal for *Bs*LOV and YF1, but higher for PAL. On the one hand, these findings suggest that the ISC quantum yield is influenced by the immediate environment of the chromophore. On the other hand, this reveals a variation in ISC efficiency among different LOV domains. This could result from an enhanced rate of ISC, potentially caused by a stronger heavy-atom effect as a result of the sulfur of the cysteine residue being closer. However, neither the crystal structures (Fig. 1) nor the very similar and even somewhat slower decay rate of ^1^FMN* observed for PAL would support that. Hence, we suggest a decreased rate of internal conversion to the dark-adapted state in PAL as the causal root of the observation, in line with recent studies in the literature.^40^

### 4.2 Thio-adduct formation

The mechanism of adduct formation *A*_390_ in LOV domains remains an active topic of discussion in the literature. In our measurements, we observe time constants of this process of 6.1 µs for *Bs*LOV and 8.7 µs for the full-length YF1 construct, respectively. The difference in these timescales could be attributed to additional conformational changes present in the multidomain architecture of YF1 or constraints imposed on the LOV domain by the attached Jα-helix and the kinase module, which may constitute an additional kinetic barrier, either in parallel to or sequential with the adduct formation step. Comparison to reported values for *Bs*LOV or full-length YtvA measured in H_2_O (1.6 to 2.0 µs)^32–34^ reveals a consistent kinetic isotope effect (KIE), a phenomenon that has been previously noted for YtvA^28^ and for other LOV domains.^41^ The presence of a KIE typically indicates that a proton or hydrogen atom transfer is the rate-limiting step in the reaction mechanism. While this mechanistic step appears conserved, variations in overall reaction rates between different constructs likely reflect contributions from domain-specific conformational dynamics superimposed on the fundamental photochemical process.

### 4.3 YF1 and *Bs*LOV

The YF1-minus-*Bs*LOV double-difference spectra not only disentangle the response of the effector domain from that of the photosensory LOV domain, but also reveal that the Jα-helix and effector domain exert an influence on the LOV domain itself. Although the differences before the formation of *A*_390_ remain small, no respective scaling of the YF1 and *Bs*LOV spectra could be found, which diminished the remaining bands to noise level in the resulting double-difference spectrum. The residual signal might arise from coupling of the FMN to the A′α-/Jα-helix dipoles or from the A′α-/Jα-helices exerting a steric influence on the LOV domain, influencing the binding pocket to a certain degree. Nonetheless, the signals at early times cancel out to a high degree upon subtraction.

Upon formation of the *A*_390_ state, however, the overall double-difference band intensities increase significantly, reflecting the strong impact of the effector and linker domains on the secondary structure rearrangements upon photoactivation. These might entail both, changes in the LOV domain embedded in the full-length construct as compared with the free LOV domain and the alterations in the linker and effector domains during signal propagation. Since the former are expected to be relatively small and considering the significant alterations in quaternary structure reported for YF1^13^ it appears evident that the observed amide bands are dominated by downstream signaling. We suggest that the bleach/transient pair at ≈1632(−)/1643(+) cm^−1^ are arising from the supercoiling of the Jα-helices observed by Berntsson *et al.*^13^ This is supported by the frequency observed for the Jα-helices of *As*LOV2 in D_2_O (1629 cm^−1^).^31^ Further, this is in line with the expected blue-shift upon supercoiling of α-helices, arising from the decreased solvent accessibility of the backbone CO groups.^42^ Features observed around 1620 and 1680 cm^−1^ might indicate structural adaptions involving β-sheet elements outside of the LOV domain. Since all non-LOV β-sheets are localized in the CA domain, this suggests a rearrangement involving these subdomains already upon formation of the *A*_390_ state. In any case, global structural changes in YF1 already appear concurrently with the adduct formation, in line with reported X-ray scattering experiments on *Bs*LOV and YF1.^13,43^

Previous studies have shown that variations in Jα linker length modulate the photoregulation of kinase activity: constructs with 7n repeat linkers display light-repressed activity, whereas those containing an additional residue (7*n* + 1) exhibit light-enhanced activity.^44^ The latter has been interpreted as a register shift in the coiled coil, altering the relative angular orientation between domains and thereby governing catalytic output.^14^ Based on this, the experimentally observed^13,14^ left-handed supercoiling that remodels the angular orientation has been proposed^14^ as the underlying mechanism for light-dependent repression of kinase activity in YF1. Since these angular changes occur concurrently with adduct formation, YF1 may already transition toward phosphatase activity at this early stage of the photocycle.

### 4.4 Signal propagation in PAL

Expression of the isolated *Nm*LOV domain was not successful. Therefore, it remains challenging to distinguish whether the observed signals in the EADS associated with *A*_390_ (blue line in Fig. 4C) originate from changes within the LOV domain itself or from structural rearrangements in the effector domains of PAL. Nevertheless, the bleach observed at 1629 cm^−1^ closely corresponds to the signal previously assigned to the Jα-helices in YF1, suggesting a tentative analogous assignment in PAL. This feature could reflect structural changes occuring in the Jα-helices relaying the signal to the ANTAR domain, consistent with the proposed structural mechanism underlying autoinhibition of RNA binding in the dark-adapted state.^10^ PAL thus also appears to exhibit interdomain signal propagation mediated by the Jα-helices already upon formation of the *A*_390_ intermediate. Unlike in YF1, however, the Jα-helices in PAL do not lie directly between the LOV and effector domains but instead transmit the signal through a network of non-covalent contacts.

### 4.5 The signaling state

Previous X-ray solution scattering experiments have reported a further structural adaptation in YF1 with a time constant of ≈250 ms.^13^ We did not observe that kinetic step by transient IR spectroscopy, neither in the YF1 data (Fig. 3), nor in the YF1-*Bs*Lov double-difference spectrum (Fig. 5). The latter should be more sensitive to detect small changes in the effector domain, as the dominant contribution from the LOV domain is subtracted out. The structure sensitivity of the amide I band originates from two effects: intra-molecular hydrogen-bonding between peptide units in α-helices and β-sheets, as well as dipole–dipole coupling between adjacent peptide bonds. In ref. 13, it was concluded that the CA subdomains move relative to the DHp helices, but stay intact as secondary structure motif. While such an arrangement might slightly change the inter-helix coupling, it is known that this has a very weak effect on the amide I band, and it is conceivable that transient IR spectroscopy is essentially blind to that type of structural modification.

Conversely, this implies that the structural changes in PAL are more severe, possibly involving the unfolding or refolding of some of the secondary structure motifs of the effector domain. Such processes will generate a detectable response in the amide I spectrum. In fact, the additional kinetic step in PAL is characterized mainly by spectral changes in the regions around ≈1625 and ≈1675 cm^−1^. These regions have been associated to β-sheet and turn elements.^45,46^ A tentative assignment could thus be adaptations in the N-terminal PAS domain of PAL.

### 4.6 N-terminal *versus* C-terminal signaling

With the multidomain constructs YF1 and PAL, we chose two bacterial representatives from the wide field of LOV regulated proteins mainly due to the respective N- and C-terminal placement of the effector domains. The aim was to elucidate the respective signal propagation from the photosensory to the signal transducer module by studying potential differences in the amide mode evolution. Up to the formation of *A*_390_, the PAL and YF1 LOV domains exhibit comparable photochemistry. This is not surprising, considering the high similarity of the LOV domains governing early reaction steps. However, with the formation of the thio-adduct, the parallels between the constructs diminish, as a comparison between the emerging amide I mode changes shows (see Fig. 4). Most strikingly, PAL undergoes an additional conformational transition on the seconds time scale before reverting to the dark-adapted state, a process that is not observed in YF1. This observation indicates that C- and N-terminal effector domain coupling in LOV-based systems involves fundamentally different signal transduction mechanisms. While the secondary structural changes in the YF1 activation pathway have already been characterized by X-ray solution scattering experiments,^13^ we propose here a larger structural change for PAL. That is, supercoiling of the Jα-helices directly induces a rotation of the histidine kinase domains in YF1, leading to the sequestration of the active-site histidine residues.^47^ In contrast, we propose that the signal is likewise transmitted *via* Jα-helices in PAL, but reaches the ANTAR domain *via* noncovalent interactions. Due to this indirect mode of signal transmission, an additional structural rearrangement, which is slower, is required to complete the transition to the final light-adapted state. The pivotal, yet versatile, role of the Jα-helix in LOV signaling is in line with previously identified mechanisms of similar systems such as *As*LOV2, where a sequence of adduct formation, Jα-helix unfolding, and subsequent dimerization are key steps of the signal transmission.^48–51^ However, in contrast to *As*LOV2, we observe structural changes involving the Jα-helices already upon formation of the thioadduct, and complete unfolding of the Jα-helices does not occur as part of the signal transduction in the multidomain proteins YF1 and PAL.

### 4.7 Recovery of the dark-adapted state

The decay of the light-adapted state has previously been found to display a KIE in a related system,^3^ and has also been reported to proceed *via* a base-catalyzed mechanism.^52^ These observations suggest that the rate-limiting step in the thermal recovery process likely involves a proton transfer rather than a hydrogen atom transfer, in line with the catalytic role of a base in facilitating deprotonation. Notably, very different time constants for the back-reaction in H_2_O have been reported for YF1 and YtvA, ranging from 16 minutes to over 100 minutes,^12,13,33^ making it impossible to directly infer the presence or magnitude of a KIE in our system based solely on available lifetimes. Moreover, this pronounced variability highlights the sensitivity of the recovery kinetics to minor differences in experimental conditions, such as pH, buffer composition, and temperature, factors that can exert a substantial influence on the reaction rate.

## Conclusion

In conclusion, this study advances our understanding of signal propagation mechanisms in FMN-binding LOV-based photoreceptors by resolving the complete photocycle dynamics in two distinct multidomain proteins. Through the use of time-resolved infrared spectroscopy and the strategic decoupling of photosensor and effector domains, we demonstrated that in YF1, signal transduction to the effector domain occurs concurrently with adduct formation, without evidence of further structural adaptation at later timescales. This finding contrasts with earlier X-ray solution scattering data suggesting a conformational process on the order of 250 ms.

Notably, our investigation of the natural multidomain photoreceptor PAL revealed an additional kinetic phase on the seconds timescale. While the precise structural correlations remain to be elucidated, we propose that this late process reflects an indirect propagation of the signal *via* the Jα-helix to downstream effector domains – a mechanism not previously resolved in time-resolved studies of LOV proteins. This divergence in kinetic behavior between YF1 and PAL underscores how domain architecture and interdomain coupling critically shape signaling pathways within multidomain photoreceptors.

Taken together, these findings not only refine mechanistic models of LOV domain signaling but also inform the future design of optogenetic tools, where the timing and separation of signal propagation steps can be crucial parameters for biological control. Future studies should aim to directly visualize the structural transitions associated with the late kinetic phase in PAL, potentially through time-resolved structural techniques such as transient X-ray solution scattering or ultrafast serial crystallography. Additionally, investigating how these mechanisms translate into functional outputs in cellular contexts would provide valuable insights into the physiological relevance of these photophysical processes.

## Author contributions

P. H., J. S., and I. S. conceived the study. R. E. H., P. J., P. F., and P. J. H. performed spectroscopic research. P. J., S. J. H., M. M., F. S., and P. J. H. performed biochemical research. C. W., P. N., and I. S. performed computational research. R. E. H., P. F., and P. J. analyzed the experimental data. P. H. designed and built the spectroscopic setup. P. H., J. S., and I. S. were in charge of funding and the research infrastructure. P. H. supervised the project. R. E. H. prepared the figures. R. E. H., P. F., and C. W. wrote the original draft. R. E. H., P. H., I. S., P. J., and P. J. H. edited and reviewed the paper with contributions from all authors.

## Conflicts of interest

There are no conflicts to declare.

## Supplementary Material

CP-028-D5CP03982G-s001

## Data Availability

The data that support the findings of this study are openly available in ZENODO (https://zenodo.org/records/18186126). Supplementary information is available. See DOI: https://doi.org/10.1039/d5cp03982g. Supplementary data has been deposited with Protein Data Bank.^9,10^
