# Supplementary material for: Signal propagation in LOV-based multidomain proteins: time-resolved infrared spectroscopy reveals the complete photocycle of YF1 and PAL
Source: Phys Chem Chem Phys. 2026 Jan 8;28(4):2847–57. doi: 10.1039/d5cp03982g (PMC12797116; doi:10.1039/d5cp03982g)
Supplement: CP-028-D5CP03982G-s001 [file CP-028-D5CP03982G-s001.pdf]

Supplementary Information (SI) for Physical Chemistry Chemical Physics.
This journal is © the Owner Societies 2026

Supporting Information

Signal Propagation in LOV-Based Multidomain Proteins: Time-Resolved Infrared Spectroscopy Reveals the Complete Photocycle of YF1 and PAL

Raoul E. Herzog,[*‡a] Philipp Janke,[‡a] Paul M. Fischer,[a] Philipp J. Heckmeier,[a] Chongyao Wei,[bc] Probal Nag,[bc] Sina J. Hartmann,[d] Matthias Mulder,[d] Fabienne Stierli,[d] Jörg Standfuss,[ad] Igor Schapiro,[*bce] and Peter Hamm[*a]

[a] *Department of Chemistry, University of Zurich, Winterthurerstrasse 190, CH-8057 Zurich, Switzerland*

[b] *Fritz Haber Center for Molecular Dynamics Research Institute of Chemistry, The Hebrew University of Jerusalem, Jerusalem 9190401, Israel*

[c] *Department of Physics, TU Dortmund University, Otto-Hahn-Str. 4, DE-44227 Dortmund, Germany*

[d] *Laboratory of Biomedical Research, PSI Center for Life Sciences, Forschungsstrasse 111, CH-5232 Villigen PSI, Switzerland*

[e] *Research Center Chemical Sciences and Sustainability, University Alliance Ruhr, DE-44801 Bochum, Germany*

‡ These authors contributed equally to this work.

Corresponding authors: raoul.herzog@chem.uzh.ch, peter.hamm@chem.uzh.ch, igor.schapiro@tu-dortmund.de.

Sequences

```
BsLOV   ------------------------------------------------------------------------------------------------------------------------  0
YF1     ------------------------------------------------------------------------------------------------------------------------  0
PAL     MKVNRPAERASFGSFVLDAGSARFVGSDELALVLGFAPGDVVLTPAVVLAHLHPDDRLEWQAGLQRCLATGRPVVVNHLLLTAEAEPRPAMTTLTALTEQDRVRAVTGVITDLSDRVRRA  120

BsLOV   --------------------------------------------------------------------------------------------------------------------MHHH  4
YF1     ---------------------------------------------------------------------------------------------MASFQSFGIPGQLE---------VIKK  18
PAL     TEAEIRQAVRAAAATRSEIDQAKGIVMAAFDVDADQAFALLKWHSSQSNRKLRDLATGMIEGLAAANSALPLRRRLSTVFTDMGCPAPSTKGWTVPVTDIGLPPTSGLIPTALLPGILTR  240

BsLOV   HHHHGGSGVVITDPALEDNPIVYVNQGFVQMTGYETEEILGKNCRFLQGKHTDPAEVDNIRTALQNKEPVTVQIQNYKKDGTMFWNELNIDPMEIED--KTYFVGIQNDIT---------  113
YF1     ALDHVRVGVVITDPALEDNPIVYVNQGFVQMTGYETEEILGKNCRFLQGKHTDPAEVDNIRTALQNKEPVTVQIQNYKKDGTMFWNELNIDPMEIED--KTYFVGIQNDITEHQQTQARL  136
PAL     AAHDASVAITVADVTAPDQPLVYANPAFERLTGYAAAEVLGRNCRFLQAESGDPHERSAIRSAIANGDAVTTLIRNFRQDGHAFWNEFHLSPVRNGAGRVTHYIGYQLDVTERVERDQQL  360

BsLOV   ------------------------------------------------------------------------------------------------------------------------  113
YF1     QELQSELVHVSRLSAMGEMASALAHELNQPLAAISNYMKGSRRLLAGSSDPNTPKVESALDRAAEQALRAGQIIRRLRDFVARGESEKRVESLSKLIEEAGALGLAGAREQNVQLRFSLD  256
PAL     EQLASLEHHHHHH-----------------------------------------------------------------------------------------------------------  373

BsLOV   ------------------------------------------------------------------------------------------------------------------------  113
YF1     PGADLVLADRVQIQQVLVNLFRNALEAMAQSQRRELVVTNTPAADDMIEVEVSDTGSGFQDDVIPNLFQTFFTTKDTGMGVGLSISRSIIEAHGGRMWAESNASGGATFRFTLPAADENL  376
PAL     ------------------------------------------------------------------------------------------------------------------------  373

BsLOV   ---------  113
YF1     EHHHHHHHH  385
PAL     ---------  373
```

Fig. S1 Sequential alignment of *Bs*LOV, YF1, and PAL. Bold: conserved residues for all three proteins. Purple: β-sheet. Orange: α-helical. Marked in red: F328 (PAL) and the corresponding position in BsLOV and YF1.

Global Fitting

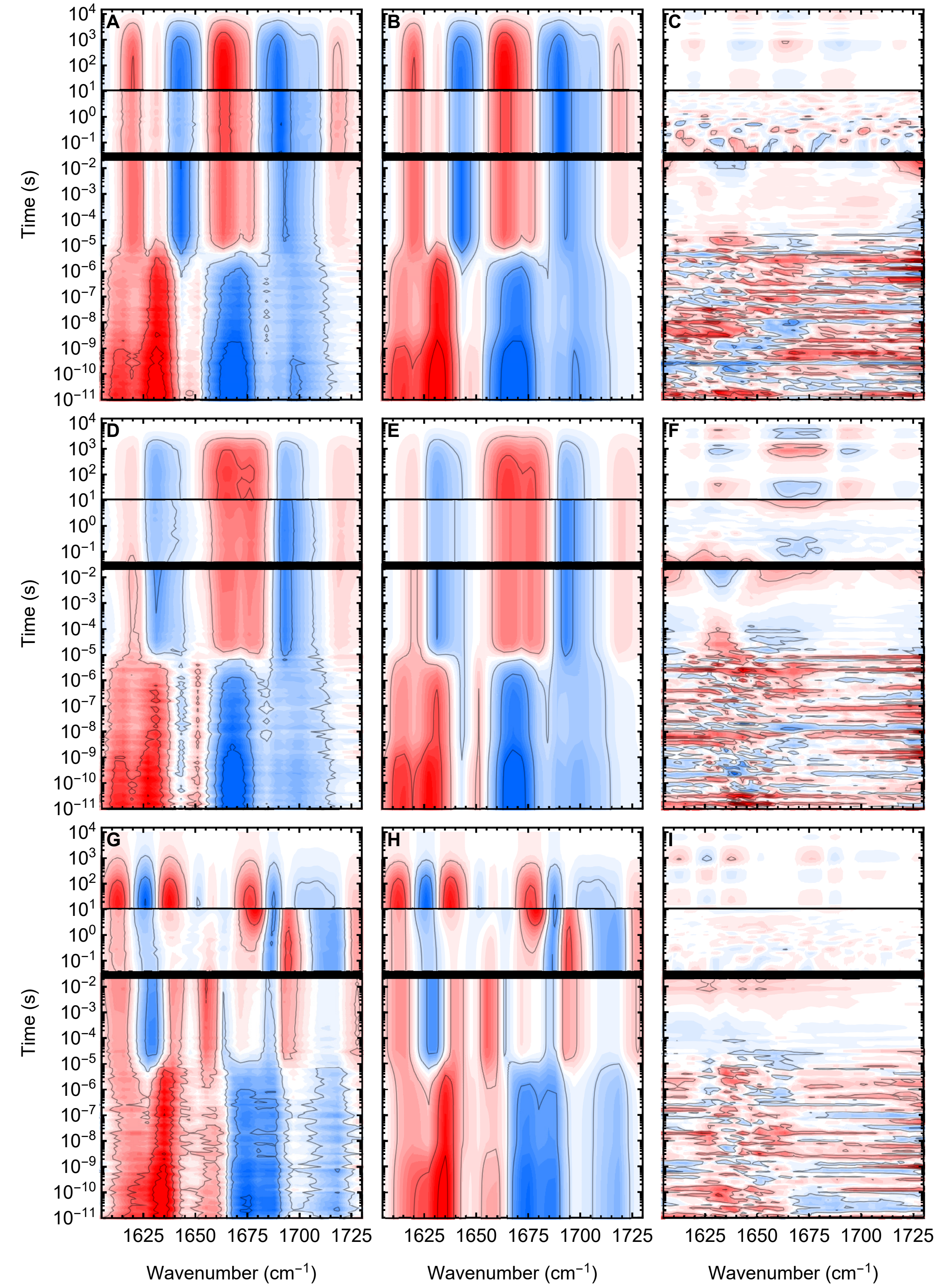

Fig. S2 Contour plots of the experimental TR-IR spectra (A,D,G), the respective global fits (B,E,H), and the fitting residuals (C,F,I) of *Bs*LOV (A-C), YF1 (D-F), and PAL (G-I).

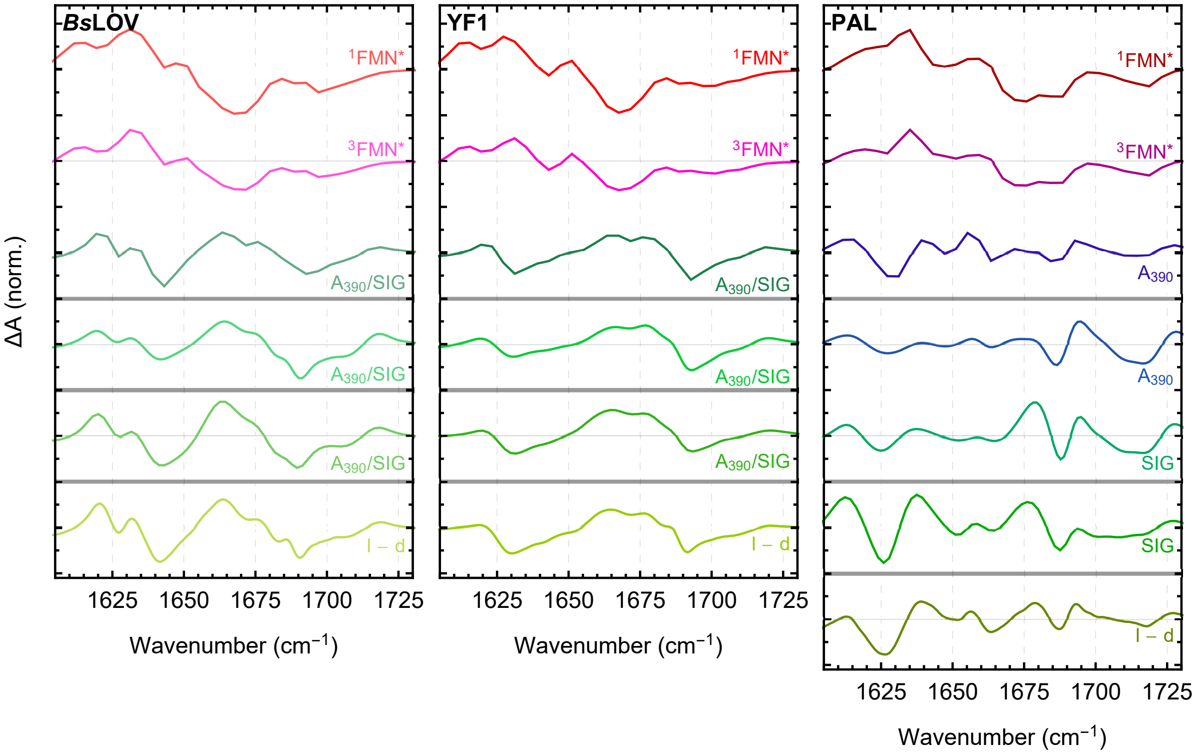

Fig. S3 EADS obtained from global fits to the TR-IR data. Thick gray lines represent the cut between the individually recorded and fitted time-windows. The EADS directly before and after each gray line represent an EADS associated with the same photophysical state.

Setup

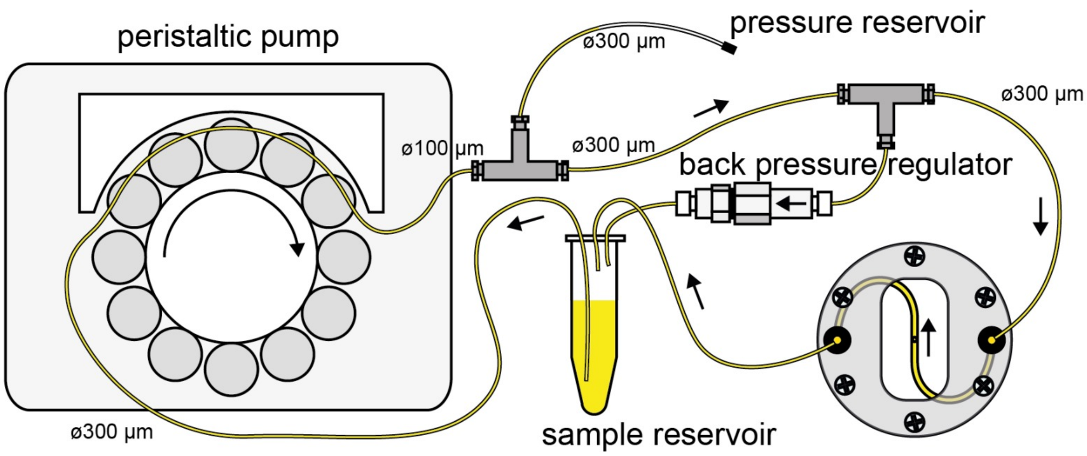

Fig. S4 Schematic illustration of the stop-flow sample-delivery system as adapted from Ref 1 with the additional flowpath through a back-pressure regulator.

Hybrid QM/MM Simulations

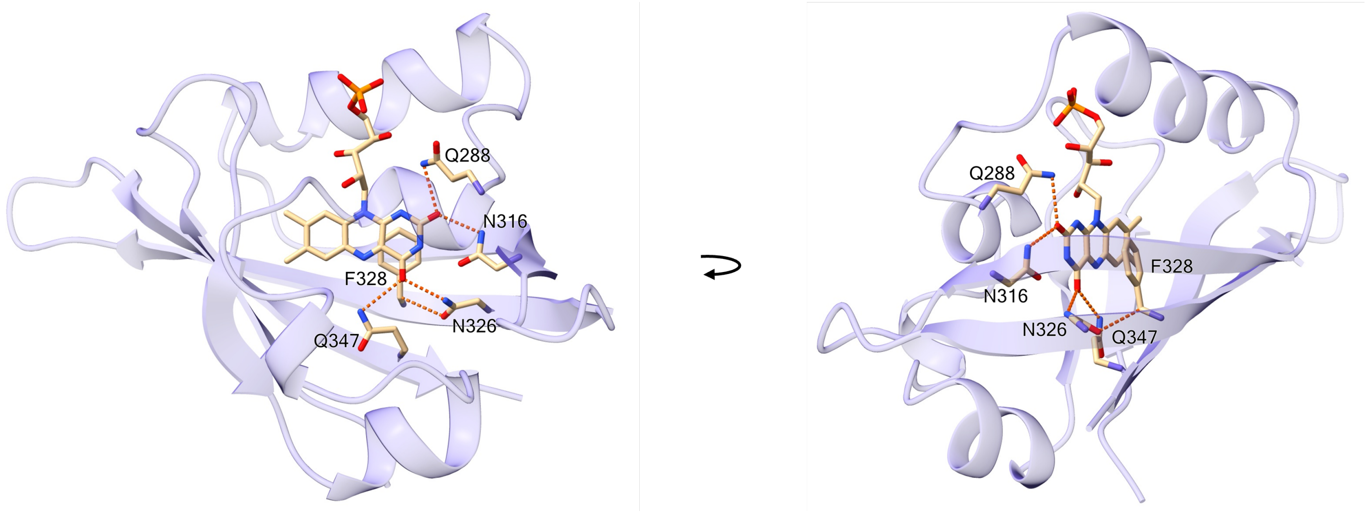

Fig. S5 The QM-MM partitioning chosen for *Nm*LOV in two different views. The carbon atoms in the QM region are marked in beige, while the carbon atoms outside the QM region are marked in purple.

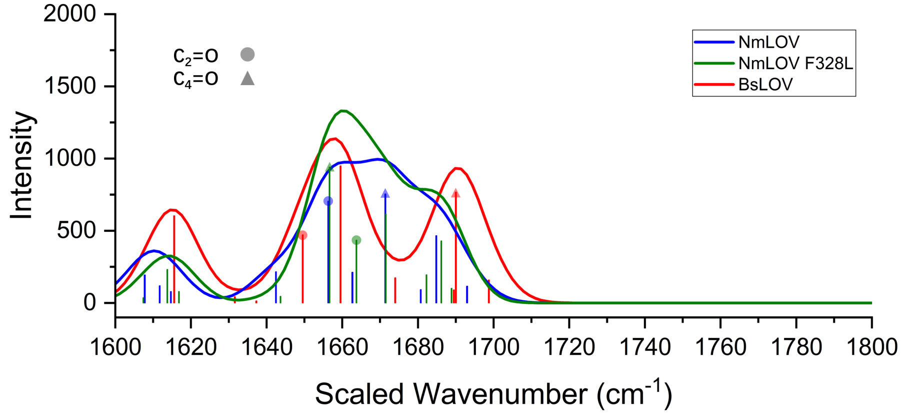

Fig. S6 The calculated QM/MM IR spectra of *Nm*LOV, its F328L mutant and *Bs*LOV, computed at B3LYP-D3BJ/6-311G* level of theory. The purpose of the mutation was to test the influence of the F328's sidechain on the C2=O and C4=O vibrations. The vibrations of C2=O and C4=O are highlighted by circles and triangles, respectively, on top of the sticks.

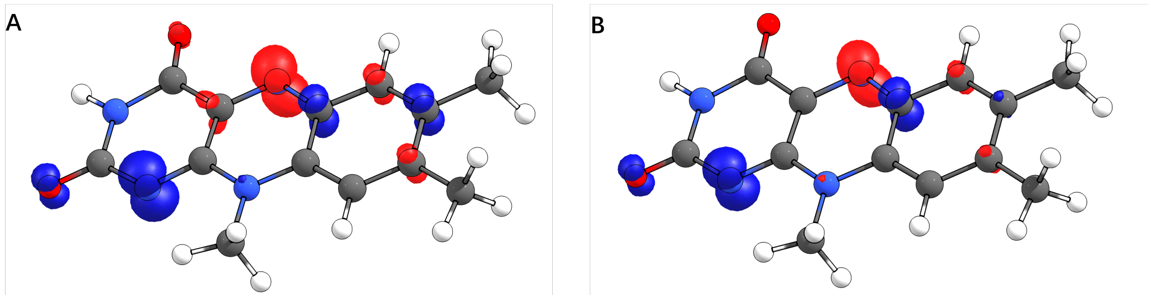

Fig. S7 Electron density difference maps of lumiflavin. (A) $S_1 - S_0$; (B) $T_1 - S_0$. Calculations were performed at the S_0-optimized geometry at the CAM-B3LYP/6-311G* level of theory. Regions of electron density depletion and accumulation are indicated in blue and red, respectively.

References

1 D. Buhrke, J. Ruf, P. Heckmeier and P. Hamm, *Rev. Sci. Instrum.*, 2021, 92, 123001.
